# Multifactorial construction of low‐grade and high‐grade endometrial cancer recurrence prediction models

**DOI:** 10.1002/ijgo.70031

**Published:** 2025-02-25

**Authors:** Yachai Li, Jia Yan, Yuanmei Deng, Peixuan Wang, Xue Bai, Wei Qin

**Affiliations:** ^1^ Department of Gynecology Affiliated Hospital of Heibei University Baoding China; ^2^ Department of Obstetrics and Gynecology Affiliated Hospital of North China University of Science and Technology Tangshan China; ^3^ Hebei University Baoding China; ^4^ Department of Integrative Traditional Chinese and Western Medicine Affiliated Hospital of Heibei University Baoding China

**Keywords:** endometrial cancer, immunohistochemical markers, nomogram, prediction model, recurrence‐free survival

## Abstract

**Objective:**

To analyze independent risk factors for endometrial cancer (EC), a common female cancer globally, and construct individualized prediction models for EC recurrence.

**Methods:**

The EC patients from the medical record system were divided into low‐grade (*n* = 392) and high‐grade (*n* = 183) groups. Immunohistochemical expression of estrogen receptor, progestin receptor, Ki67, and L1 cell adhesion molecule (L1CAM) was detected. Univariate Cox regression, LASSO regression, and stepwise Cox regression were applied for identifying independent risk factors for EC recurrence. The predictive value of the model was verified by using receiver operating characteristics curves, bootstrap method, calibration curves, and decision curve analysis curves.

**Results:**

Multivariate Cox analysis revealed that FIGO (the International Federation of Gynecology & Obstetrics) Stage, progestin receptor, lymphovascular space invasion (LVSI), and tumor size were independent risk factors for low‐grade EC recurrence‐free survival (RFS), and FIGO Stage, L1CAM, LVSI, and pelvic lymph node status were independent risk factors for high‐grade EC. The areas under the curves at 1‐, 3‐, and 5‐year RFS in low‐grade and high‐grade groups were 0.881/0.825, 0.888/0.853, and 0.807/0.832, respectively. Calibration curves were close to the diagonal, and the decision curve analysis curves were located mostly above the All and None lines in both groups.

**Conclusion:**

The prediction model demonstrates accurate discriminative ability and strong calibration capability. It has high clinical application value and provides decision making information regarding RFS for both low‐grade and high‐grade EC patients. This may assist in formulating personalized treatment plans, monitoring follow‐up strategies, and implementing lifestyle intervention measures.

## 1 INTRODUCTION

Endometrial cancer (EC) is one of the major reproductive system tumors affecting numerous women worldwide,^1^ with 417 000 new cases and 97 000 deaths reported globally in 2020.^2^ Immunohistochemistry is a relatively simple and well‐established technique with moderate costs, easily carried out by pathologists. Studies have identified a variety of proteins associated with the recurrence and metastasis of EC, including estrogen receptor (ER), progestin receptor (PR), Ki67, and L1 cell adhesion molecule (L1CAM), all of which may serve as valuable prognostic markers. Identifying specific markers associated with EC recurrence, in combination with clinical and pathologic indicators, can enhance the accuracy of prognosis assessments for EC patients. This study aims to combine immunohistochemical indicators with pathologic parameters to establish a rapid and accurate prediction model. Currently, logistic regression models or Cox proportional hazards models are commonly used to predict disease risks and model disease risks. However, these methods involve complex formulas and time‐consuming calculations in clinical practice. To predict the probability of a patient experiencing a particular outcome event at a future time point, the use of nomogram models is concise, clear, and easy to understand.

The present study seeks to leverage immunohistochemical markers in EC patients, combined with clinical data and pathologic features, to separately establish predictive models for disease recurrence risk and prognosis in low‐grade and high‐grade EC. These models aim to guide the selection of postoperative adjuvant treatment strategies for EC, assess the risk of recurrence and prognosis, and provide guidance for personalized treatment.

## 2 MATERIALS AND METHODS

### 2.1 Patients

Inclusion criteria for the study were: (1) patients initially treated with surgery for disease that was confirmed by pathologic examination of postoperative specimens as EC between January 2017 and December 2019 in the Affiliated Hospital of Hebei University; (2) patients with complete clinical data with a postoperative follow‐up time of 3 years or longer; and (3) informed consent was obtained from all participants.

Women were excluded if they had the presence of malignant tumors in other systems or had received neoadjuvant treatments such as chemotherapy or hormone therapy.

### 2.2 Data

Basic patient data, including age, height, weight, body mass index (BMI, calculated as weight in kilograms divided by the square of height in meters), hypertension (based on the 1999 World Health Organization diagnostic criteria^3^), diabetes (based on the 1999 World Health Organization diagnostic criteria^4^), other medical history, preoperative tumor marker indicators (carcinoembryonic antigen >5 μg/L, cancer antigen [CA] 125 >35 U/mL, CA199 >37 U/mL), postoperative pathology, postoperative adjuvant treatment, and immunohistochemical marker (ER, PR, Ki67, L1CAM). Additionally, the follow‐up period was calculated from the date of surgery with the end date set at December 2022. Recurrence‐free survival (RFS) referred to the time from the date of surgery to the confirmed recurrence date based on histology or imaging. All procedures were performed in compliance with relevant laws and institutional guidelines and have been approved by the Ethics Committee of the Affiliated Hospital of Hebei University.

All immunohistochemical results were selected at random in five high‐power fields (×400) under a standard optical microscope, and the positive rate of parameter results was expressed as a percentage. Staining was considered positive when brown‐yellow or brown particles appeared in the cell nucleus for PR, ER, Ki67, and in the cell cytoplasm and/or membrane for L1CAM.

### 2.3 Groups

For the purpose of this study, patients were grouped as low‐grade (G1, G2) or high‐grade (G3, serous carcinoma, clear cell carcinoma). Grade G1 means non‐squamous solid growth pattern of 5% or less, grade G2 means non‐squamous solid growth pattern from >5% to 50%, and grade G3 means non‐squamous solid growth pattern of >50%.^5^

### 2.4 Statistical analysis

Software applications included R software (version 3.6.1) and SPSS 25.0. The measurement data were presented as mean ± standard deviation, and analyzed for intergroup differences using independent sample *t* tests. The enumeration data were presented as number (%) and analyzed for intergroup differences using *χ* ^2^ test. The abnormally distributed continuous variables were presented as the median (interquartile range). A *P* value of 0.05 or less indicated a statistically significant difference. Variables with a *P* value less than 0.05 in the univariate analysis were in involved in multivariate Cox analysis. The receiver operating characteristics (ROC) curve was used to evaluate the effectiveness of the prediction model and the areas under curves (AUC) of ROC were calculated to measure the model's discriminatory ability.

## 3 RESULTS

### 3.1 Baseline characteristics of low‐grade and high‐grade groups

A total of 392 cases in the low‐grade group and 138 cases in the high‐grade group were included in the analysis of general clinical data, serologic examination indicators, and pathologic results, including: age, BMI, hypertension, menopausal status, FIGO (the International Federation of Gynecology & Obstetrics) Stage, ER, PR, Ki67, L1CAM, tumor size, lymphovascular space invasion (LVSI), cervical stromal infiltration, involvement of the lower uterine segment, myometrial invasion, pelvic lymph nodes, para‐aortic lymph nodes, adjuvant treatment, and other relevant baseline characteristics (Tables 1, 2, 3).

**TABLE 1:** Basic characteristics for two groups.^a^

Characteristics	Low‐grade group (*n* = 392)	High‐grade group (*n* = 138)
Age, years	55.00 (50.00–62.00)	59.00 (55.00–65.00)
BMI	27.23 (25.42–29.64)	26.04 (24.83–27.91)
Diabetes		
Yes	84 (21.43)	32 (23.19)
No	308 (78.57)	106 (76.81)
Hypertension		
Yes	189 (48.21)	80 (57.97)
No	203 (51.79)	58 (42.03)
Heart disease		
Yes	35 (8.93)	18 (13.04)
No	357 (91.07)	120 (86.96)
Other comorbidities		
Yes	77 (19.64)	26 (18.84)
No	315 (80.36)	112 (81.16)
Menopause		
Yes	236 (60.20)	117 (84.78)
No	156 (39.80)	21 (15.22)
CEA		
≤5.00	382 (97.45)	135 (97.83)
>5.00	10 (2.55)	3 (2.17)
CA125		
≤35.00	352 (89.80)	119 (86.23)
>35.00	40 (10.20)	19 (13.77)
CA199		
≤37.00	367 (93.62)	127 (92.03)
>37.00	25 (6.38)	11 (7.97)
FIGO staging		
I	354 (90.31)	96 (70.07)
II	18 (4.59)	8 (5.84)
III	20 (5.10)	33 (24.09)
Pathologic type		
Endometrioid adenocarcinoma	393 (100.00)	62 (44.90)
Serous carcinoma	0 (0.00)	54 (39.1)
Clear cell carcinoma	0 (0.00)	19 (13.80)
Carcinosarcoma	0 (0.00)	3 (2.20)
Tumor size, cm	1.80 (1.30–3.00)	2.50 (1.50–3.50)
LVSI		
Yes	37 (9.44)	47 (34.06)
No	355 (90.56)	91 (65.94)
Cervical stroma infiltration		
Yes	21 (5.36)	20 (14.49)
No	371 (94.64)	118 (85.51)
Involvement of lower uterus segment		
Yes	80 (20.41)	42 (30.43)
No	312 (79.59)	96 (69.57)
Muscle layer infiltration		
None or ≤1/2	327 (83.42)	86 (62.32)
>1/2	65 (16.58)	52 (37.68)
Pelvic lymph node involvement		
Negative or not performed	386 (98.47)	113 (81.88)
Positive	6 (1.53)	25 (18.12)
Para‐aortic lymph node involvement		
Negative or not performed	389 (99.23)	127 (92.03)
Positive	3 (0.77)	11 (7.97)
Adjuvant therapy		
Radiotherapy	75 (19.13)	18 (13.04)
Chemotherapy	5 (1.28)	40 (28.99)
Radiotherapy and chemotherapy	44 (11.22)	59 (42.75)
None	268 (68.37)	21 (15.22)

Abbreviations: BMI, body mass index (calculated as weight in kilograms divided by the square of height in meters); CA125, cancer antigen 125; CA199, cancer antigen 199; CEA, carcinoembryonic antigen; LVSI, lymphovascular space invasion.

^a^ Data are presented as median (interquartile range) or as number (percentage).

**TABLE 2:** Immunohistochemical expression in the two groups.^a^

	Low‐grade group	High‐grade group
ER, %	80.00 (70.00–90.00)	40.00 (5.00–80.00)
PR, %	80.00 (50.00–90.00)	20.00 (0.00–60.00)
Ki67, %	30.00 (20.00–50.00)	60.00 (40.00–70.00)
L1CAM, %	10.00 (0.00–30.00)	30.00 (20.00–60.00)

Abbreviations: ER, estrogen receptor; L1CAM, L1 cell adhesion molecule; PR, progestin receptor.

^a^ Data are presented as median (interquartile range).

**TABLE 3:** Recurrence status and follow‐up period of the two groups.^a^

	Low‐grade (*n* = 392)	High‐grade (*n* = 138)	*P* value
Recurrence (yes/no)	23/369	26/112	<0.001
Follow‐up time, months	48 (41–56)	47 (39, 45)	0.019
	47.68 ± 10.33	43.58 ± 9.06	
Recurrence‐free survival time, months	23 (21–28)	15 (11–34)	0.779
	26.04 ± 6.11	24.54 ± 13.96	

^a^ Data are presented as number/total number, median (interquartile range) or as mean ± standard deviation.

### 3.2 Univariate regression analysis

A total of 25 initial variables were included in the analysis. Univariate regression analysis for the low‐grade group indicated that age, FIGO Stage, ER, PR, L1CAM, LVSI, tumor size, cervical stromal infiltration, pelvic lymph nodes, muscle layer infiltration, and para‐aortic lymph nodes may be important factors influencing RFS in the low‐grade group (Table 4). Univariate regression analysis for the high‐grade group indicated that weight, FIGO Stage, LVSI, L1CAM, muscle layer infiltration, pelvic lymph nodes, and para‐aortic lymph nodes may be important factors influencing RFS in the high‐grade group (Table 5).

**TABLE 4:** Single‐factor regression analysis in the low‐grade group.

	β	HR (95% CI)	*P* value
Age	0.049	1.05 (1.00–1.10)	0.032
Height	0.042	1.04 (0.97–1.12)	0.251
Weight	0.005	1.01 (0.97–1.04)	0.764
BMI	0.008	0.99 (0.90–1.09)	0.870
Diabetes			
Yes	—	—	0.165
No	0.849	2.34 (0.71–7.74)	
Hypertension			
Yes	—	—	0.578
No	0.212	1.24 (0.59–2.61)	
Heart disease			
Yes	—	—	0.745
No	0.239	1.27 (0.30–5.35)	
Other comorbidities			
Yes	—	—	0.810
No	0.119	1.13 (0.43–2.96)	
Menopause			
Yes	—	—	0.380
No	−0.355	0.70 (0.32–1.55)	
CEA			
≤5.00	—	—	0.684
>5.00	0.414	1.51 (0.21–11.15)	
CA125			
≤35.00	—	—	0.476
>35.00	0.385	1.47 (0.51–4.24)	
CA199			
≤37.00	—	—	0.827
>37.00	0.160	1.17 (0.28–4.95)	
FIGO stage			
I	—	—	
II	1.347	3.85 (1.12–13.21)	0.032
III	2.605	13.53 (5.96–30.72)	<0.001
ER	−0.021	0.98 (0.97–0.99)	0.003
PR	−0.024	0.98 (0.96–0.99)	<0.001
Ki67	−0.000	1.00 (0.98–1.02)	0.962
L1CAM	0.021	1.02 (1.01–1.03)	<0.001
LVSI			
Yes	—	—	<0.001
No	−1.765	0.17 (0.08–0.37)	
Tumor size	0.337	1.40 (1.27–1.55)	<0.001
Cervical stromal infiltration			
Yes	—	—	0.034
No	−1.144	0.32 (0.11–0.92)	
Involvement of the lower uterine segment			
Yes	—	—	0.114
No	−0.640	0.53 (0.24–1.17)	
Muscle layer infiltration			
<50%	—	—	0.001
≥50%	1.244	3.47 (1.62–7.41)	
Pelvic lymph node involvement			
Negative	—	—	<0.001
Positive	2.834	17.02 (5.85–49.53)	
Para‐aortic lymph node involvement			
Negative	—	—	0.007
Positive	1.843	6.32 (0.86–46.53)	
Adjuvant therapy			
R	—	—	
C	3.292	26.89 (7.92–91.35)	<0.001
R and C	0.608	1.84 (0.59–5.70)	0.292
None	−0.690	0.50 (0.19–1.36)	0.174

Abbreviations: BMI, body mass index (calculated as weight in kilograms divided by the square of height in meters); C, chemotherapy; CA, cancer antigen; CEA, carcinoembryonic antigen; CI, confidence interval; ER, estrogen receptor; HR, hazard ratio; L1CAM, L1 cell adhesion molecule; LVSI, lymphovascular space invasion; PR, progesterone receptor; R, radiotherapy.

**TABLE 5:** Single factor regression analysis in the high‐grade group.

	β	HR (95% CI)	*P* value
Age	−0.002	0.998 (0.946–1.053)	0.949
Height	−0.035	0.966 (0.895–1.043)	0.375
Weight	−0.042	0.958 (0.920–0.998)	0.040
BMI	−0.112	0.894 (0.798–1.002)	0.053
Diabetes			
Yes	—	—	
No	0.438	1.549 (0.536–4.480)	0.419
Hypertension			
Yes	—	—	
No	0.032	1.032 (0.495–2.152)	0.932
Heart disease			
Yes	—	—	
No	0.411	1.508 (0.455–4.992)	0.501
Other comorbidities			
Yes	—	—	
No	−0.679	0.507 (0.222–1.158)	0.107
Menopause			
Yes	—	—	
No	0.017	1.017 (0.349–2.965)	0.975
CEA			
≤5.00	—	—	
>5.00	−16.039	0.913 (0.842–3.712)	0.997
CA125			
≤35.00	—	—	
>35.00	0.577	1.782 (0.724–4.383)	0.209
CA199			
≤37.00	—	—	
>37.00	0.939	2.558 (0.881–7.422)	0.084
FIGO stage			
I	—	—	
II	0.575	1.778 (0.218–14.472)	0.591
III	2.358	10.569 (4.481–24.931)	<0.001
ER	−0.009	0.991 (0.980–1.003)	0.141
PR	−0.002	0.998 (0.987–1.010)	0.762
Ki67	0.004	1.004 (0.987–1.021)	0.653
L1CAM	0.023	1.023 (1.012–1.034)	<0.001
LVSI			
Yes	—	—	
No	−398	0.247 (0.112–0.545)	0.001
Tumor size	−0.025	0.975 (0.817–1.164)	0.778
Cervical stromal infiltration			
Yes	—	—	
No	−0.705	0.494 (0.217–1.123)	0.092
Involvement of the lower uterine segment			
Yes	—	—	
No	−0.690	0.502 (0.241–1.044)	0.065
Muscle infiltration			
<50%	—	—	
≥50%	1.208	3.348 (1.578–7.100)	0.002
Pelvic lymph node involvement			
Negative	—	—	
Positive	1.860	6.423 (3.083–13.382)	<0.001
Para‐aortic lymph node involvement			
Negative	—	—	
Positive	1.451	4.268 (1.886–9.654)	<0.001
Adjuvant therapy			
R	—	—	
C	1.384	3.992 (0.511–31.219)	0.187
R and C	1.366	3.921 (0.512–30.044)	0.189
None	0.809	2.245 (0.233–21.647)	0.484

Abbreviations: BMI, body mass index (calculated as weight in kilograms divided by the square of height in meters); C, chemotherapy; CA, cancer antigen; CEA, carcinoembryonic antigen; CI, confidence interval; ER, estrogen receptor; HR, hazard ratio; L1CAM, L1 cell adhesion molecule; LVSI, lymphovascular space invasion; PR, progesterone receptor; R, radiotherapy.

### 3.3 LASSO regression

**FIGURE 1:**
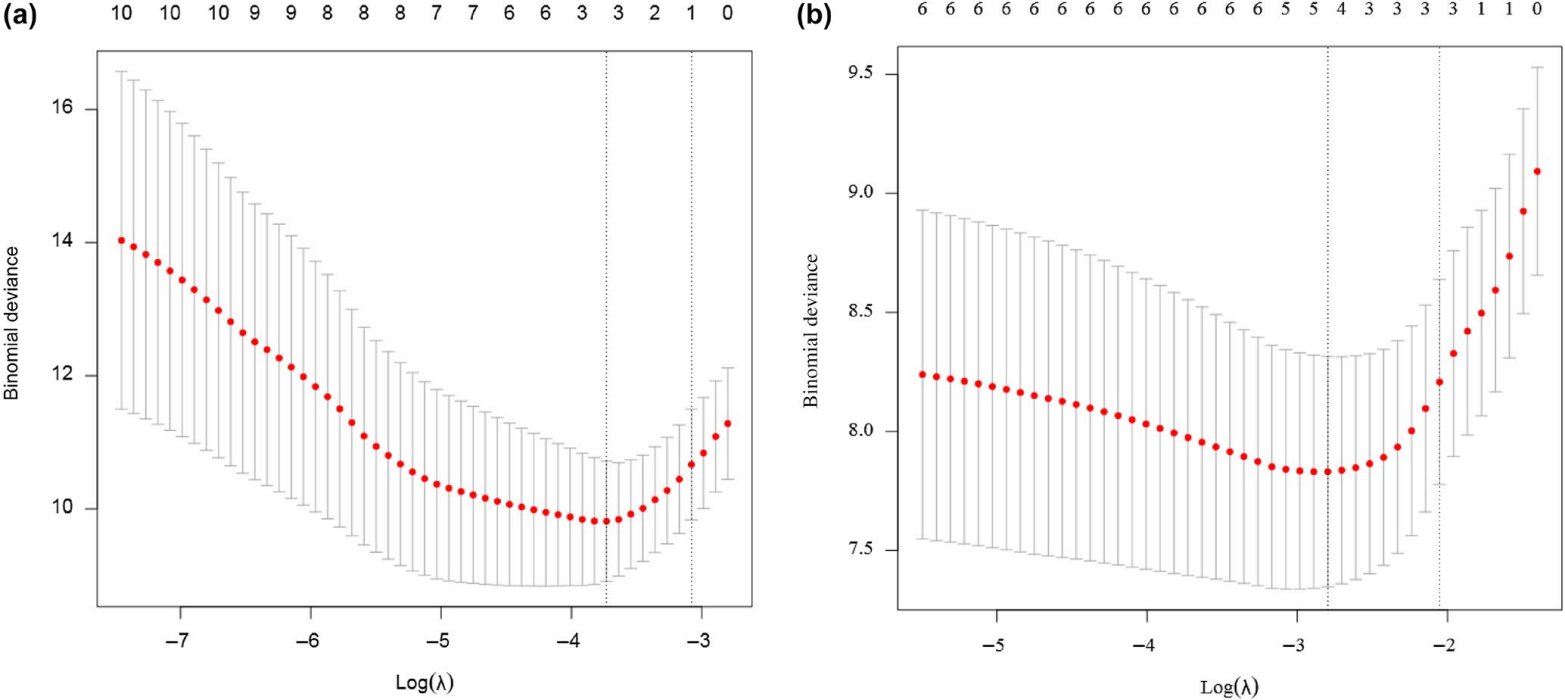
LASSO regression cross‐validation. (a) Low‐grade group; (b) high‐grade group. LASSO regression analysis was performed on the variables selected from single‐factor Cox regression analysis, and the non‐zero coefficient variables were identified as potential risk factors.

The variables selected through single factor Cox regression analysis in both groups were subjected to LASSO regression analysis. The non‐zero coefficient variables obtained from this analysis were considered potential risk factors. For the low‐grade group, the selected variables were FIGO Stage, PR, LVSI, tumor size, and pelvic lymph node involvement. For the high‐grade group, the selected variables were FIGO Stage, L1CAM, LVSI, muscle infiltration, and pelvic lymph node involvement (Table 6). The results of the LASSO regression cross‐validation are shown in Figure 1.

**TABLE 6:** LASSO regression analysis.

Low‐grade group		High‐grade group	
Variables	Coefficient	Variables	Coefficient
FIGO stage	0.80075053	FIGO stage	0.69337209
PR	−0.0059298	L1CAM expression	0.01469556
LVSI	−0.20119682	LVSI	−0.1076105
Tumor size	0.26561326	Muscle layer infiltration	0.01602169
Pelvic lymph node involvement	0.40983255	Pelvic lymph node involvement	0.41354406

Abbreviations: FIGO, the International Federation of Gynecology & Obstetrics; L1CAM, L1 cell adhesion molecule; LVSI, lymphovascular space invasion; PR, progesterone receptor.

### 3.4 Construction of nomogram

**FIGURE 2:**
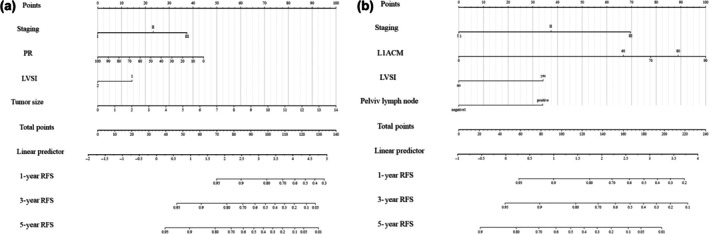
Line chart predictive model. (a) Low‐grade group; (b) high‐grade group. The variables selected by regression analysis were used as independent influencing factors to establish a column chart of endometrial cancer recurrence risk, thereby calculating the predictive probability of endometrial cancer recurrence. Each independent line segment represents different influencing factors, and the length represents the score. The sum of the scores of each line segment is the total score of the patient, and the endometrial cancer recurrence rate is positively correlated with the total score. L1ACM, L1 cell adhesion molecule; LVSI, lymphovascular space invasion; PR, progestin receptor; RFS, recurrence‐free survival.

Using the “rms” package in R software, the variables selected from the regression analysis were considered as independent influencing factors to construct a nomogram for predicting the risk of EC recurrence. (Figure 2).

### 3.5 Evaluation and validation

**FIGURE 3:**
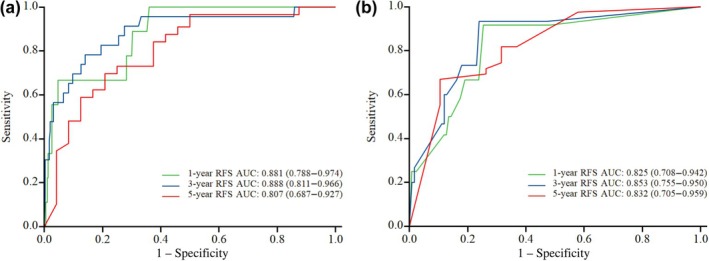
Area under the receiver‐operating characteristics curve (AUC). (a) Low‐grade group; (b) high‐grade group. To reflect the predictive performance of the model, the overall predictive ability of the model was evaluated using AUC. AUC = 0.5 represents complete inconsistency, indicating that the model has no predictive function. AUC = 1 represents completely consistent, indicating that the model is completely consistent with reality. AUC between 0.5 and 0.7 is considered low discrimination, AUC between 0.71 and 0.9 is considered moderate discrimination, and AUC above 0.9 is considered high discrimination. In this study, all the AUC in the two groups were close to 0.9. RFS, recurrence‐free survival.

**FIGURE 4:**
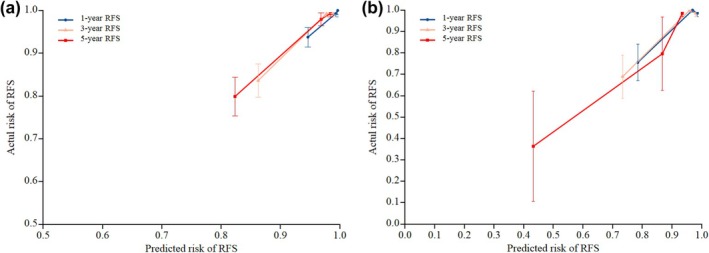
Calibration plot. (a) Low‐grade group; (b) high‐grade group. The calibration degree of the prediction model is shown by a calibration curve. The standard curve is a straight line with a slope of 1 passing through the origin of the coordinate axis. The closer the calibration curve is to the standard curve, the better the predictive ability of the column chart. The calibration curve shows that the predicted curve of the model is close to the ideal curve, indicating that the predicted risk of endometrial cancer recurrence by the model is consistent with the actual risk, and the accuracy of the model is high. If the predicted value equals the actual value, the curve completely overlaps with the diagonal. If the predicted value is higher than the actual value, the curve is above the diagonal and indicates an overestimation of the risk. If the predicted value is less than the actual value, the curve is below the diagonal and indicates underestimation of the risk. This study showed the calibration curve close to the standard curve, which meant the high accuracy of the model. The calibration plot was generated using bootstrap resampling with 1000 iterations. For each iteration, the model was refitted and predicted probabilities were calculated. The plot displays the average predicted probabilities against the observed outcomes, with error bars representing the 95% confidence intervals. RFS, recurrence‐free survival.

**FIGURE 5:**
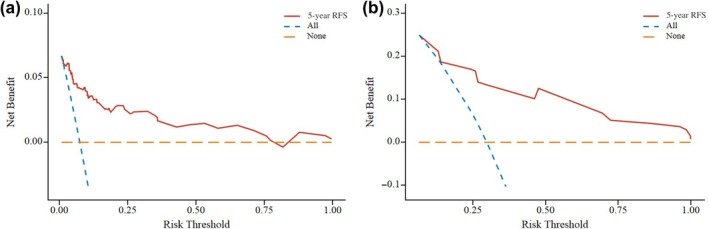
Clinical decision curve analysis curve. (a) Low‐grade group; (b) high‐grade group. The clinical decision curve is used to evaluate the clinical usability of predictive models. When all people are patients and have not received clinical intervention, the net benefit is represented by the None line. When all patients have received clinical intervention, the net benefit is represented by the All line. When the curves are located in the upper right corner of the All and None lines, it indicates that the predictive model has good clinical value. RFS, recurrence‐free survival.

To reflect the predictive performance of the model, the overall predictive ability of the model was assessed using the AUC. For the low‐grade group, the AUC was 0.881 for 1‐year RFS, 0.888 for 3‐year RFS, and 0.807 for 5‐year RFS. In the high‐grade group, the AUC was 0.825 for 1‐year RFS, 0.853 for 3‐year RFS, and 0.832 for 5‐year RFS (Figure 3). The calibration accuracy of the prediction model was demonstrated using the calibration curve. Bootstrap resampling was performed 1000 times, and the essence of this curve lies in comparing the actual risk with the predicted risk for 1, 3, and 5 years. The calibration curves are presented in Figure 4, and the decision curve analysis curves are shown in Figure 5.

## 4 DISCUSSION

Despite the generally favorable prognosis of EC under appropriate treatment, about one‐third of these patients experience distant metastasis.^6^ Women with EC in the present study comprised two patient groups with distinct risk factors and long‐term prognoses.

Research indicates that acquired factors such as obesity, diabetes, and metabolic syndrome are closely associated with histologic grading, not only promoting the pathogenesis of the disease but also correlating with adverse outcomes.^7^ Estrogen, a known endometrial growth factor, is synthesized and released by the ovaries before menopause whereas postmenopause, the adipose tissue becomes the primary source of estrogen.^8^ Hence, obesity is considered closely related to EC. Obesity‐related insulin resistance is also a key factor associated with EC, and insulin resistance can lead to diabetes. Obesity and hypertension contribute to the development of insulin resistance, which can lead to type 2 diabetes and promote the occurrence and progression of malignant tumors through mechanisms such as hyperinsulinemia and activation of the insulin‐like growth factor‐1 signaling pathway.^9^

In this study, BMI and hypertension showed differences between the low‐grade and high‐grade groups, but the multifactorial analysis did not reveal a correlation between obesity, hypertension, diabetes, and EC recurrence. However, these three factors are still recognized as the triad of EC, requiring patient education to actively control weight, blood pressure, and blood sugar.

High expression of ER and PR is directly related to less myometrial invasion and a lower incidence of lymph node metastasis in patients with EC. PR has two subtypes, PRα and PRβ, but the exact biologic or clinical differences between these two PR subtypes remain largely unknown. Compared with the primary tumor, the expression of ER and PRα is significantly decreased in recurrent lesions. The decrease in PRα expression and its gene promoter methylation may play a role in tumor progression. ER and PR status are independent prognostic factors for patients, particularly in estrogen‐dependent cancers such as breast cancer and EC. The loss of ER expression is associated with tumor progression in estrogen‐dependent cancers and may also influence tumor behavior in other cancers, such as ovarian cancer. The loss of PR expression, especially PRα, plays a crucial role in prognosis and recurrence, correlating with a poorer prognosis for disease‐free survival. In this study, PR was identified as an independent risk factor for the low‐grade group, with decreased expression associated with higher recurrence risk.

L1CAM is essentially a 200‐ to 220‐kDa transmembrane glycoprotein.^10^ Initially identified as a neuronal cell adhesion molecule in the central nervous system, L1CAM expression has also been found in other cell types, where it is believed to promote rapid proliferation, invasion, and metastasis of tumor cells,^11^ partly through the activation of the extracellular signal‐regulated kinase pathway, inducing the expression of genes associated with motility and invasion. Positive L1CAM expression has been associated with an increased rate of distant recurrence in EC,^12^ particularly in type II EC.^13^ In this study, L1CAM analysis between the low‐grade and high‐grade groups showed no significant statistical significance, possibly because of the small sample sizes. Immunohistochemical analysis of 865 tumor samples from the PORTEC‐1 and PORTEC‐2 trials showed a significant increase in the risk of distant recurrence and pelvic lymph node recurrence with high L1CAM expression.^14^ The present study found that L1CAM was an independent risk factor for recurrence in the high‐grade group and was associated with adverse prognosis.

Some published studies suggest that tumor size is not related to the risk of recurrence in women with EC,^15^ whereas others believe that tumor size appears to be an important risk factor for EC recurrence.^16^, ^17^ A meta‐analysis of 40 articles and 53 276 EC patients showed that tumor size greater than 2 cm increased the risk of recurrence for FIGO Stage Ia EC, and tumor size greater than 2 cm significantly predicted a higher recurrence rate for FIGO Stages I–III EC.^18^ Çakır et al.^19^ showed that the risk of recurrence was higher in patients with tumor diameter greater than 2 cm, about 2.45 times higher than in patients with tumor diameter of 2 cm or less (odds ratio 2.45; 95% confidence interval 1.73–3.48; *P* < 0.001). The present study revealed an association between tumor diameter and recurrence in EC, with no correlation with the recurrence risk of other pathologic types of EC. LVSI is a manifestation in pathology defined as the presence of tumor cells in lymphatic vessels or small blood vessels outside the main tumor. Tortorella et al.^20^ also found that patients with LVSI had a significantly higher risk of disease recurrence and death, with extensive LVSI being the strongest independent factor for recurrence in multivariate analysis.

Compared with patients without LVSI, those with LVSI had a higher 5‐year recurrence rate and the strongest independent prognostic factors for pelvic regional recurrence, distant metastasis, and overall survival.^21^, ^22^, ^23^ Our study found that LVSI is an independent risk factor for EC recurrence, consistent with previous research.

The external iliac lymph nodes are the most common site of pelvic lymph node metastasis in EC patients. Univariate analysis in some studies showed that age over 50 years, adnexal metastasis, and pelvic lymph node metastasis were associated with recurrence. Polterauer et al.^24^ reported that the proportion of positive pelvic lymph nodes was related to progression‐free survival and overall survival. In a study by Tangjitgamol et al.^25^ involving 82 patients, positive pelvic lymph node ratio was one of the significant adverse prognostic factors for progression‐free survival through univariate analysis. Pelvic lymph node positivity increases the risk of recurrence and reduces overall survival. For pathologic Stage III C EC confined to pelvic lymph nodes, postoperative pelvic radiotherapy is a feasible treatment option. In this study, RFS in the high‐grade group was related to pelvic lymph node status, and postoperative radiotherapy may be considered for patients with lymph node positivity.

Using statistical principles, a column chart was constructed based on the results of a complex regression equation, and the chart was then used to visualize the results. Compared with traditional TNM staging, column charts are considered superior in many cancers and have even been proposed as an alternative or new standard. One of the main advantages is that individualized risk estimates can be calculated based on patient and disease characteristics.

In this study, independent risk factors for postoperative RFS in EC were plotted into a column chart model to predict RFS at 1, 3, and 5 years for EC patients. Independent risk factors for low‐grade EC and high‐grade EC differed, requiring separate models for prognosis, treatment planning, and counseling.

Model predictive performance was evaluated using the AUC, with values ranging from 0.5 to 1, positively correlating with predictive ability. An AUC of 0.5 indicates complete inconsistency, suggesting that the model has no predictive effect, while an AUC of 1 indicates complete consistency, indicating that the model is entirely consistent with reality. Previous studies defined lower discrimination as an AUC between 0.50 and 0.70, moderate discrimination as an AUC between 0.71 and 0.90, and high discrimination as an AUC above 0.90. The AUC values for the predictive models in this study were all greater than 0.71, indicating good predictive performance.

The standard curve is theoretically a curve that passes through the origin of the coordinate axis and has a slope of 1, and if the calibration curve is closer to the standard curve, then the predictive ability of the column chart is better. The calibration curve for this model shows that the predictive curve is close to the ideal curve, suggesting that the model's prediction of the risk of EC recurrence is relatively consistent with the actual risk, and the model has high accuracy. The predicted 1‐, 3‐, and 5‐year RFS for both groups showed small differences from the actual RFS, and the calibration curve was close to the diagonal, indicating high predictive accuracy.

The decision curve analysis curves in this study were generally located above the diagonal of both the All line and the None line, indicating that the constructed predictive model had high clinical utility. This visual column chart predictive model, based on pathologic features and immunohistochemical markers, can assist clinical doctors in decision making, providing a better risk assessment for recurrence in the two patient groups and helping to select individualized treatment plans. This study used internal validation methods to validate the model, and the results showed that the model had good applicability and predictive performance. However, decisions regarding the application and selection of postoperative adjuvant treatment for patients still need to be made by clinical doctors based on the patients' conditions.^26^

In conclusion, this study found that FIGO Stage, PR, LVSI, and tumor diameter were independent prognostic factors for RFS in low‐grade EC. FIGO Stage, L1CAM, LVSI, and pelvic lymph nodes were independent prognostic factors for RFS in high‐grade EC. A predictive model of EC recurrence was constructed, which has good discriminant ability, calibration ability and clinical application value, and can be used for postoperative follow‐up decisions.

## AUTHOR CONTRIBUTIONS

YL designed, planned, and conducted the study and wrote the manuscript; JY and YD planned and conducted the study; PW planned the study and analyzed the data; XB conducted the study; and WQ designed, planned, and conducted the study and reviewed the manuscript.

## CONFLICT OF INTEREST STATEMENT

The authors have no conflicts of interest.

## Data Availability

The data that support the findings of this study are available from the corresponding author upon reasonable request.
